# Rehabilitation Towards Functional Independence in a Patient With Metastatic Carcinoma of Lung and Paraplegia: A Case Report

**DOI:** 10.7759/cureus.50675

**Published:** 2023-12-17

**Authors:** Aditi M Akhuj, Tejaswini Fating

**Affiliations:** 1 Department of Community Health Physiotherapy, Ravi Nair Physiotherapy College, Datta Meghe Institute of Higher Education and Research, Wardha, IND

**Keywords:** carcinoma of lung, chemotherapy, squamous cell carcinoma, paraplegia, case report, rehabilitation, physiotherapy, lung cancer

## Abstract

Lung cancer metastasis is a complex process. This case report describes a case of a 58-year-old man with carcinoma of the lung with bony metastasis to spine T9-T11 with the chief complaints of inability to move both lower limbs, breathlessness, and difficulty in bed mobility. Motor impairments may transpire in three different forms, which are paraplegia, hemiplegia, or quadriplegia. Functional electrical stimulation (FES) with body weight support treadmill training (BWSTT) is a widespread rehabilitation approach used to restore motor function of the lower limb and balance. This case report specifies the physiotherapeutic rehabilitation protocol, which includes dyspnea management, FES with BWSTT, proprioceptive neuromuscular facilitation, etc. for a patient undergoing physiotherapy The patient’s occupational requirements and enhancement in executing daily living tasks were the focus of the physiotherapeutic rehabilitation. The outcomes used were the modified Medical Research Council (mMRC) grading of dyspnea and the Functional Independence Measure (FIM). We report a marked increment in muscle tone and strength, active range of motion (AROM), and significant enhancement in the individual’s functional independence with physiotherapeutic protocol.

## Introduction

Squamous cell carcinoma (SCC) is the most prevalent type of epithelial cancer that can spread metastatically, affecting multiple anatomic sites [1]. Lung cancer continues to be the most often diagnosed disease worldwide and is the leading cause of cancer-related fatalities, carrying an enormous burden on the world's health system [2]. When lung carcinomas are identified, they are usually at stage IV of metastatic spread. The most typical symptoms include dyspnea, coughing, loss of weight, exhaustion, and anxiety [3]. Smoking is the leading cause of death for males with lung cancer, and it is also becoming increasingly prevalent in women. Bone is the earliest location of metastases from lung cancer. Approximately 20-30% of lung cancer patients already have bone metastasis during diagnosis, and 30-70% of bone metastases are linked to lung cancer. Hypercalcemia, instability in the spine, pathological fractures, bone pain, and spinal cord compression are all possible effects of bone metastases. The majority of the time, bone metastases are advanced and have a negative impact on the patient's quality of life (QOL) [4].

The most often metastasized sites from advanced non-small cell lung cancer (NSCLC) are the vertebral bones, which have a poor prognosis, particularly in cases of pathological fractures and spinal cord compression [5]. Patients with carcinoma of the lung may receive chemotherapy, radiation, and surgery as treatment options. Even if they are likely life-saving, they are linked to several often reported and related symptoms, including dyspnea, tiredness, and depression, which can be severe and long-lasting. As the paraplegic ages and experiences physiological and psychological issues, concerns about their QOL arise. Paraplegia is the term used to describe paralysis of both lower limbs in the absence of upper limb involvement [6]. Physical therapy is among the most crucial components of spinal cord injury (SCI) rehabilitation, which includes interventions associated with participation, activity limitation, and body structure and function [7].

Physiotherapy targets dyspnea management, enhancing the function of upper and lower extremities, balance, etc., by using a treatment like breathing exercises, dyspnea relieving positions, strengthening exercises, proprioceptive neuromuscular facilitation (PNF), functional electrical stimulation with body-weight-supported treadmill training (FES with BWSTT) for a patient undergoing physiotherapy. FES is intended to help individuals with central paralysis enhance the structural integrity of their lower motor neurons. FES, in conjunction with BWSTT, is one beneficial strategy for improving lower limb function and balance. It has a positive and lasting effect on the voluntary control of the patient, balance, posture, and gait. This treatment also assists the patient in reorganizing and relearning the function of limbs which are paralyzed [8]. There is currently a lack of studies demonstrating a treatment for patients undergoing chemotherapy for lung cancer. As a result, we established a training programme to help these patients manage dyspnea and anxiety and improve their motor control of lower limbs, balance, and functional independence.

## Case presentation

Patient information

A 58-year-old male, working as a labourer, who has right-hand dominance, was brought to Acharya Vinoba Bhave Rural Hospital (AVBRH), Wardha, Maharashtra, India. The patient was alright till two months back when he experienced pain in the chest region on the right side, which was gradual in onset, so he visited a local hospital, where medications like analgesics (tablet tramadol, tablet morphine) and anti-inflammatory drugs (injection hydrocort) were given, but he got no symptomatic relief. He also complained of weakness and a tingling sensation in his right foot, which radiated to the calf region. So he visited a private hospital where the patient underwent certain investigations like a contrast-enhanced CT (CECT) chest, abdomen, and pelvis, an MRI spine, a pelvic bone lesion biopsy, and a cytology exam and was diagnosed with stage IV metastatic SCC of the lung. He was referred to AVBRH for chemotherapy and underwent two cycles of chemotherapy. The Eastern Cooperative Oncology Group (ECOG) Performance Status at the time of admission was symptomatic, <50% in bed during the day. The regimen used was paclitaxel (135 mg/m2) plus carboplatin (AUC 5). On August 21, 2023, his bilateral lower limbs got paralysed, and post-chemotherapy, he started complaining of breathlessness, generalized weakness, inability to move both lower limbs, and difficulty in bed mobility. He had no complaints of any of the associated illnesses like hypertension, diabetes mellitus, or tuberculosis. He reported a history of smoking (20 packs per year) and tobacco spanning twenty years and fifteen years, respectively. On August 22, 2023, physiotherapy rehabilitation was commenced with the proper tailor-made protocol for the patient. 

Clinical findings

Before the commencement of the examination, informed consent was taken from the patient, and he was examined. On examination, he was conscious, cooperative, and well-oriented to person, place, and time. The patient was afebrile and hemodynamically stable. The patient was seen in a supine lying posture with head end elevated to 30 degrees. He was ectomorphic, with a BMI of 19 kg/m^2^. During a neurological evaluation, superficial and deep sensations were absent. Both the lower limbs were flaccid. All deep tendon reflexes were diminished (Table 1), upper limb strength was 3 out of 5 (Table 2), and the modified Medical Research Council (mMRC) grading was grade 4 (too dyspneic to leave the house or breathless when dressing). The patient was unable to stand and walk. On auscultation, air entry was reduced bilaterally. As evaluated by the Functional Independence Measure (FIM), the patient necessitated maximal assistance with basic activities of daily living (ADLs) like eating, bathing, toileting, and transferring, as well as instrumental ADLs (transportation and handling).

**Table 1:** Pre-intervention deep tendon reflexes +: Diminished reflex, ++: Normal reflex, +++: Exaggerated reflex

Reflexes	Right	Left
Bicep jerk	+	+
Triceps jerk	+	+
Knee jerk	+	+
Ankle jerk	+	+

**Table 2:** Pre-intervention muscle strength on the Manual Muscle Testing (MMT) scale 0: No contraction, 1: Flickering contraction, 2: Full Range of Motion (ROM) with gravity eliminated, 3: Full ROM against gravity, 4: Full ROM against gravity, moderate resistance, 5: Full ROM against gravity, maximal resistance

Muscles	Right	Left
Shoulder flexors	3/5	3/5
Shoulder extensors	3/5	3/5
Shoulder abductors	3/5	3/5
Elbow flexors	3/5	3/5

Diagnostic assessment

**Figure 1:**
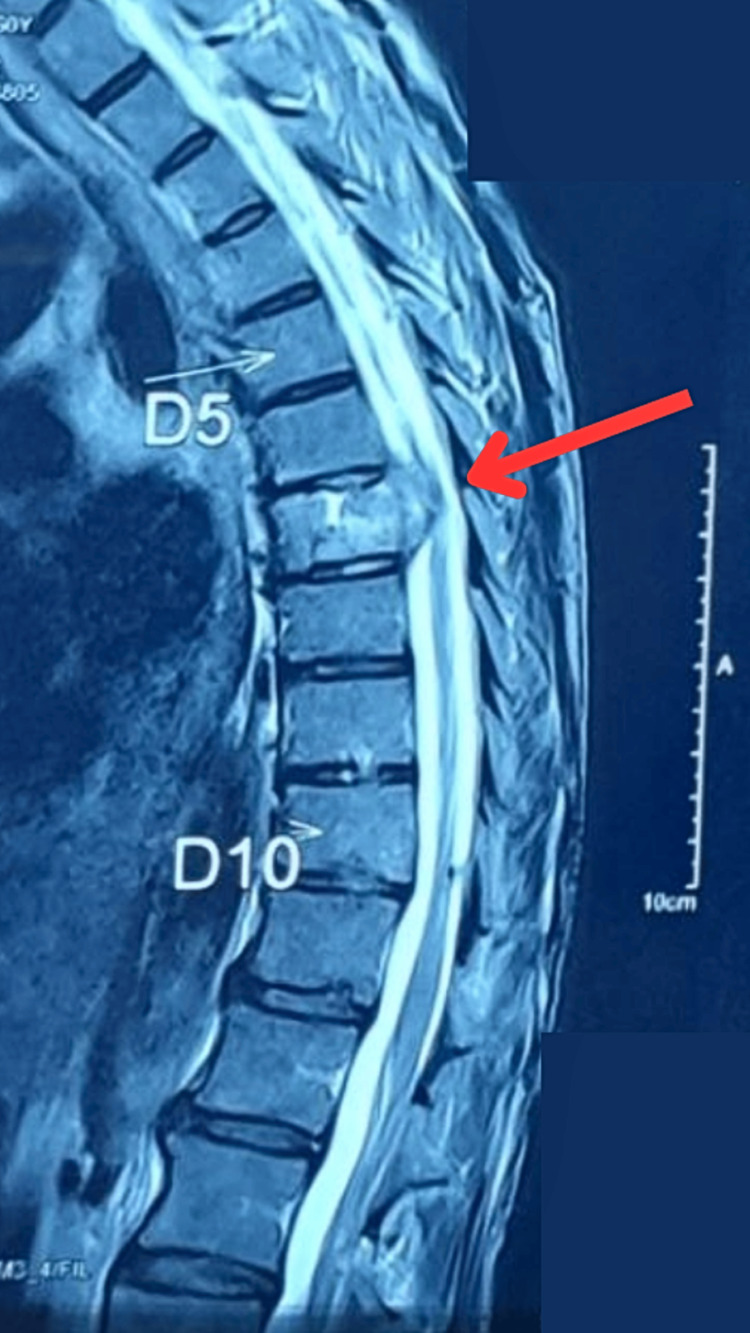
MRI of dorsal spine The red arrow points to the reduced height of the D7 vertebra and retro-vertebral anterior thecal sac, with the abutment of the spinal cord.

A complete blood count (CBC) was done. In this investigation, an increase in levels of WBCs was found. Cytology report revealed cells showing degenerative changes comprising lymphocytes and neutrophils. Pelvis bone lesion biopsy (microscopy) was done, where histological examination showed metastatic SCC. The CECT Scan of the chest, abdomen, and pelvis was performed. Subtle circumferential mural thickening was seen involving the distal oesophageal and gastro-oesophageal junction; atelectatic and fibrotic changes in the right lower and upper lobes of the lung, respectively, were noted. Osteolytic lesions were seen involving T9 and T11 vertebral bodies in a posterior element of the L2 vertebral body. These findings were suggestive of metastatic deposits. MRI of the dorsal spine (Figure 1) with whole spine screening suggested abnormal short tau inversion recovery (STIR) hyperintensity involving D7 and D9-D11 vertebra with reduced height of D7 vertebra. There was evidence of resultant focal with retro-vertebral anterior epidural collection at the D7 level, causing effacement of the anterior thecal sac with the abutment of the spinal cord, which caused spinal cord compression that resulted in paraplegia. These findings are suggestive of infective spondylodiscitis, likely Koch's.

Timeline

On August 2023, the patient was admitted to the oncology ward and underwent the first cycle of chemotherapy on August 20, 2023, and the second cycle on August 29, 2023. Physiotherapy management was started on August 21, 2023, and he received a four-week course of therapy. The patient was discharged on September 19, 2023.

Therapeutic intervention

**Figure 2:**
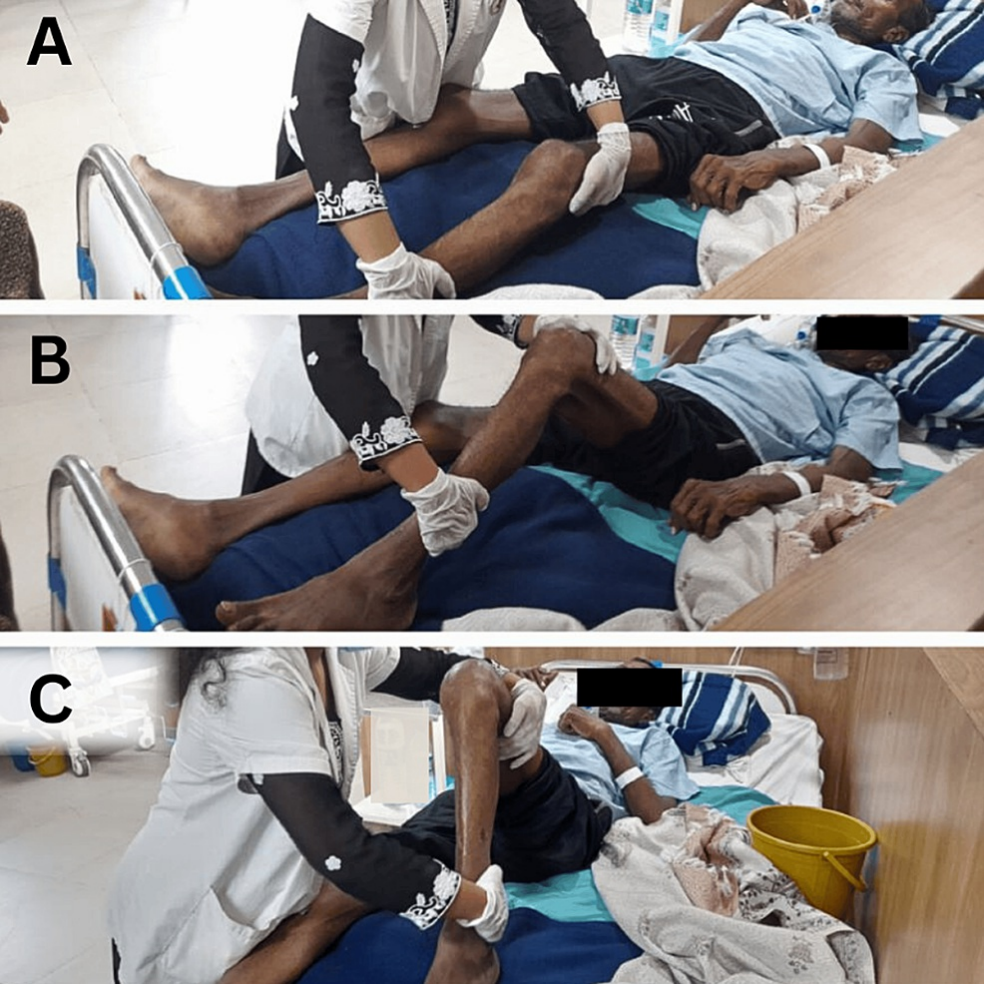
PROM exercises for lower limb PROM: passive range of motion A: Initiation of passive hip flexion, B: Mid-range of passive hip flexion, C: End-range passive hip flexion

**Figure 3:**
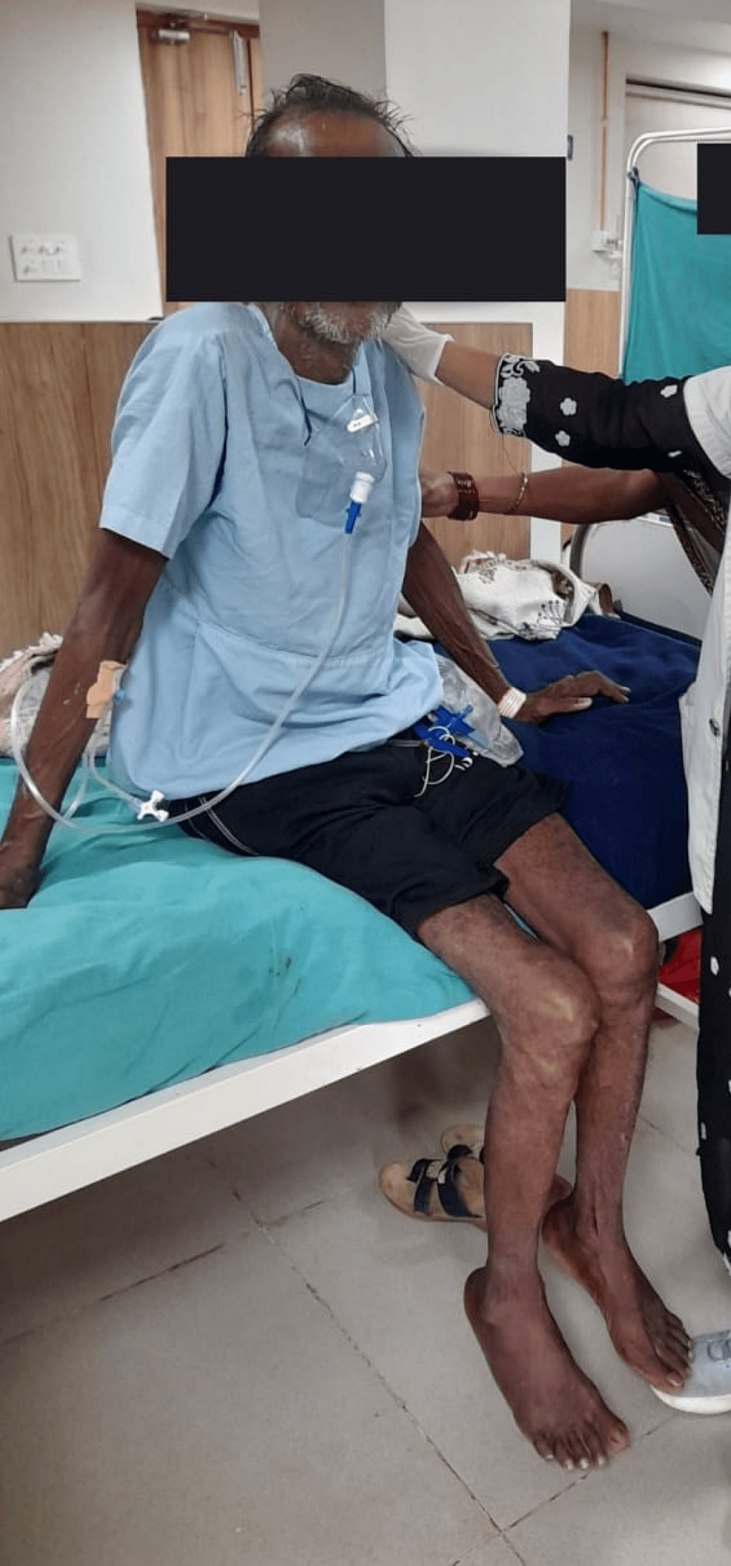
Patient sitting with minimal assistance

Physiotherapy rehabilitation protocol was planned week-wise (Table 3). Figures 2A, 2B, 2C show the therapist giving a passive range of motion to the lower limb. In Figure 3, the patient has gained sitting balance with minimal assistance.

**Table 3:** Physiotherapeutic intervention PLB: Pursed lip breathing; PROM: Passive range of motion; AROM: Active range of motion; B/L: Bilateral, UL: Upper limb; LL: Lower limb; reps: Repetitions; PNF: Proprioceptive neuromuscular facilitation; QOL: Quality of life; FES: Functional electrical stimulation; BWSTT: body-weight-supported treadmill training; N/A: Not applicable

Sr no	Problem identified	Goals of rehab	Intervention	Dosage
1.	Patient and family education	To enhance and maintain the patient’s positive attitude towards treatment for early recovery.	The patient and the family members were educated regarding the significance of exercising and how rehabilitation will help in improving his symptoms and QOL.	N/A
2.	Breathlessness	To relieve dyspnea.	Dyspnea relieving positions in side lying, sitting.	Every time the patient became dyspneic, he was asked to perform dyspnea-relieving positions.
3.	Mobility issue	To improve bed mobility.	AROM for B/L UL, PROM for B/L LL, log rolling with assistance, and bedside sitting with maximum assistance.	10 reps x 2 sets, bedside sitting for 4-5 minutes.
4.	Atonia	To build up muscular tone.	PNF rhythmic initiation to the B/L LL (D1 and D2 patterns) to enhance mobility and stability and increase the strength of LL musculature, progression to PNF slow reversals and combination of isotonic.	10 reps x 1 set.
5.	Inactivity may result in reduced strength in the UL	Maintaining upper extremity strength.	UL strengthening using a weight cuff of 1 kg.	10 reps × 2 sets, twice a day.
6.	Unable to walk	Balance and gait training	FES with BWSTT.	FES for 30 minutes, BWSTT for 20 minutes.
7.	Sensory reeducation	Sensory re-education.	Employing a variety of textures, from distal to proximal, such as cotton cloth, feathers, Turkish cloth, rubbing sand, silk cloth, etc.	10 reps × 2 sets, twice a day.
8.	Anxiety and relaxation	To improve psychological health.	Jacobson relaxation technique.	15 minutes twice a day.
9.	Secondary complications like deep vein thrombosis, bed sores, etc	To prevent secondary issues.	Ankle-toe movements, changing the position every two hours.	10 reps × 2 sets, thrice a day.
10.	Respiratory complications	To prevent respiratory problems.	Breathing exercises including diaphragmatic breathing and thoracic expansion exercises.	10 reps × 2 sets, thrice a day.

Follow-up and outcomes

An organized physical therapy interventional protocol was started. For four weeks, a follow-up was carried out once per week (Tables 4, 5). The findings of the outcome measures are shown in Table 6. A home exercise program was given, and follow-up was taken.

**Table 4:** Pre- and post-physiotherapeutic rehabilitation of muscular strength MMT: Manual muscle testing, 0: No contraction; 1: Flickering contraction; 2: Full Range of Motion (ROM) with gravity eliminated; 3: Full ROM against gravity; 4: Full ROM against gravity, moderate resistance; 5: Full ROM against gravity, maximal resistance

Muscles	Pre-treatment MMT grade	Post-treatment MMT grade	Follow-up
Upper limb			
-Shoulder flexors	3/5	4/5	4/5
-Shoulder extensors	3/5	4/5	4/5
-Shoulder abductors	3/5	4/5	4/5
-Elbow flexors	3/5	4/5	4/5
Lower limb			
-Hip flexors	0/5	2/5	2/5
-Hip extensors	0/5	2/5	2/5
-Hip abductors	0/5	2/5	2/5
-Hip adductors	0/5	2/5	2/5
-Knee flexors	0/5	2/5	2/5
-Knee extensors	0/5	2/5	2/5

**Table 5:** Pre- and post-physiotherapeutic rehabilitation tone (TGS) TGS: Tone Grading Scale, 0: No response, 1+: Decreased tone, 2+: Normal tone, 3+: Increased tone

Muscle Group	Pre-intervention	Post-intervention	Follow-up
Knee flexors	0	2+	2+
Knee extensors	0	2+	2+
Hip flexors	0	2+	2+
Hip extensors	0	2+	2+
Hip abductors	0	2+	2+
Hip adductors	0	2+	2+

**Table 6:** Pre- and post-physiotherapeutic rehabilitation outcome measures mMRC: Modified Medical Research Council; FACT-L: Functional Assessment of Cancer Therapy - Lung; BBS: Berg Balance Scale Grade 3: Stops for breath after walking 100 yards (91 m) or after a few minutes; Grade 4: Too dyspneic to leave the house or breathless when dressing

Outcome measures	Pre-intervention	Post-intervention	Follow-up
Functional Independence Measure	14/126	60/126	62/126
mMRC grading of dyspnea	Grade 4	Grade 3	Grade 3
FACT-L	13/ 28	18/28	19/28
BBS	0/56	11/56	14/56

## Discussion

Dyspnea is one of the most unsettling signs of advanced lung cancer, which can be challenging to treat. Multidisciplinary therapy is beneficial for lung cancer patients as it can improve physical functioning and quality of life while decreasing the perceived intensity of dyspnea and fatigue. Physiotherapists have an essential role in the treatment of lung cancer patients [9,10]. After a comprehensive examination, it is believed that those with advanced lung cancer can benefit from physiotherapy techniques for the management of symptoms and issues post-chemotherapy like dyspnea, lack of physical activity, fatigue associated with cancer, secretions from the lungs, depression and anxiety [11,12].

There is a lack of studies on the effects of different types of exercise interventions on dyspnea and fatigue in lung cancer patients undergoing chemotherapy despite these symptoms being commonly experienced by them. There are few studies evaluating the effect of FES with BWSTT on balance and lower limb function. One study suggested that patients who receive breathing training, which includes diaphragmatic breathing, pursed lip breathing, and stress management, observe a significant reduction in respiratory rate dyspnea and an improvement in their functional abilities [13-15]. Likewise, we gave breathing exercises and found positive results in our study. Physical, psychosocial, and environmental changes benefit dyspnea management. A review evaluated that interventions such as energy conservation techniques, usage of fans, staying in a cold room, non-invasive mechanical ventilation, anxiety-reducing techniques, supporting family or caregivers, and training have been reported as nonpharmacological methods used in dyspnea management [16].

Following treatment with the PNF technique, the patient displayed increased muscular strength and speed of movement. Cayco et al. discovered that a PNF technique combined with principles of neuroplasticity was a reliable and effective way to recover the motor outputs of elderly people who were suffering from chronic stroke. Particularly in non-spastic muscular groups, specific PNF methods can improve strength, dexterity, and speed of movement [17]. 

According to Shroff et al. [18], physical therapy helps individuals with spinal cord injuries manage their disabilities on a daily basis. It involves stimulating the muscles and nerves below the site of the injury in addition to mobilisation exercises. Bao et al. [19] and Lee et al. [20] studied the effect of FES plus BWSTT for gait rehabilitation in patients poststroke and found that it was effective in balance and function of lower limbs in patients poststroke. Similarly, we used FES plus BWSTT and got a positive effect on balance and lower limb function.

## Conclusions

Since even patients with metastatic lung cancer are living longer, competent and effectual applications must be developed at the earliest to improve the functional status and QOL of these patients. This case study reveals that well-planned physiotherapeutic intervention given for four weeks, with follow-up accompanied by FES with BWSTT and PNF approach, showed improved treatment outcomes and was endorsed to be incredibly beneficial in motor relearning and enhancing muscle tone and strength, quality of life, and functional independence in a patient with carcinoma of lung and paraplegia.
